# Informing equitable urban health policy: a multi-source geospatial assessment of spatial mismatch in medical facility distribution across Chinese cities

**DOI:** 10.3389/fpubh.2026.1775514

**Published:** 2026-02-25

**Authors:** Zeyu Zhang, Zhengchen Guo, Bingshuai Li, Lin Song

**Affiliations:** 1 School of Philosophy and Social Development, Shandong University, Jinan, China; 2 Law School, Shandong University of Technology, Zibo, China; 3 Tianjin Research Institute for Water Transport Engineering, M.O.T., Tianjin, China; 4 Department of Information Science and Technology, Dalian Maritime University, Dalian, Liaoning, China

**Keywords:** evidence-informed policy, geographically weighted regression (GWR), health equity, healthcare accessibility, medical facility distribution, public health policy, spatial mismatch, urban planning

## Abstract

**Background:**

Equitable distribution of medical facilities is a foundational element of urban health policy, particularly in rapidly urbanizing settings where spatial mismatches between healthcare supply and population demand can exacerbate health inequities. In China, despite national efforts to strengthen primary healthcare, the planning and distribution of medical facilities remain uneven, raising concerns about fairness, efficiency, and social justice in public service provision.

**Methods:**

We conducted a multi-city geospatial assessment across four major cities in Shandong Province (Jinan, Qingdao, Yantai, and Weihai) using an integrated framework that combines healthcare Points of Interest (POIs), 100-meter resolution census-based population grids, OpenStreetMap road networks, and official land use records. To evaluate spatial equity, we applied the Gini coefficient, global and local indicators of spatial autocorrelation (Moran’s I and LISA), and geographically weighted regression (GWR) to identify disparities and context-specific drivers of medical facility distribution.

**Results:**

Our analysis reveals significant over-concentration of medical resources in central urban districts, while peripheral and county-level areas face systemic under-provision. Gini coefficients ranged from 0.59 to 0.73 indicating high levels of intra-urban inequity. GWR results further show that in core areas, facility location aligns with population density and economic activity, whereas in outlying regions, inadequate transport infrastructure and inflexible land-use regulations constrain equitable access. Notably, Qixia, Liuhe, and Rongcheng emerged as critical underserved zones requiring targeted policy intervention.

**Conclusion:**

This study provides actionable, spatially explicit evidence for urban health policymakers seeking to advance equity in medical resource allocation. By linking fine-grained geospatial analytics with principles of spatial justice, our findings support the redesign of medical facility planning guidelines, the integration of accessibility metrics into smart city governance, and the prioritization of underserved areas in future health infrastructure investment. The methodological approach offers a scalable model for evidence-informed public health policy in other emerging urban contexts.

## 1 Introduction

The spatial distribution of medical resources and the issue of its equity have long been a key research focus in the fields of public health, urban planning, and social governance (1). It also serves as an important indicator for measuring the maturity of regional medical service systems and the level of social fairness (2, 3). The rational allocation of medical resources is not only directly related to the protection of residents’ health and well-being but also reflects social justice, coordinated regional development, and the overall level of social welfare at a macro level. Although equitable access to healthcare is universally recognized as a basic human right by the international community, the realization of this right still faces severe challenges in developing countries with weak medical systems (4). In recent years, with the continuous advancement of China’s urbanization process and increasing population mobility, the issue of uneven spatial allocation of medical resources has become increasingly prominent, especially in areas with significant differences in city size (5), urban–rural regions (6), and economic development levels (7).

In terms of specific spatial patterns, this typically manifests as a high concentration of medical resources in the core urban areas of eastern cities, where the number of medical facilities, medical standards, and service capacity are all at relatively high levels (8, 9). In contrast, urban–rural fringes and rural areas face issues such as insufficient numbers of medical facilities, lower levels of medical services, and poor accessibility (10). This spatially uneven distribution not only affects residents’ equitable opportunities to access medical services, leading to widening disparities in health benefits, but also further triggers a series of derivative social problems, such as excessive concentration of medical resources, overloaded operation of medical institutions in core urban areas, and weakened functions of the primary healthcare system. Increased cross-regional patient flow and insufficient primary health care have also become urgent practical challenges for China’s medical service system (11, 12).

In response to this issue, the national level has recently introduced a series of policies and reform measures to promote the balanced allocation of medical resources. For example, by implementing a tiered diagnosis and treatment system, promoting the construction of medical consortia and alliances, and strengthening the capacity of primary healthcare services, efforts are made to alleviate the problems of “difficulty and high cost in accessing medical services” and to achieve the rational flow and effective utilization of high-quality medical resources. However, the implementation effects of existing policies vary significantly across different regions, especially in the economically developed eastern coastal areas, where the distribution of medical resources among regions still shows obvious uneven characteristics. Taking Shandong Province as an example, as a major coastal economic province in China, its cities exhibit considerable differences in economic development level, population size, and infrastructure construction, making the uneven spatial distribution of medical resources particularly prominent. Provincial capital Jinan and coastal central city Qingdao, as regions with high concentration of economic, population, educational, and medical resources within the province, possess high-quality medical facilities and abundant health resources. In contrast, other prefecture-level cities, especially Yantai and Weihai, have relatively fewer medical resources, insufficient capacity in the primary healthcare system, and pronounced issues regarding the accessibility and equity of medical services between urban and rural areas and within cities. This phenomenon not only affects the health level of local residents but also poses challenges to the coordinated development of the regional medical service system.

When analyzing the spatial distribution of medical resources, sociological and geographical theories provide important interpretive perspectives. Spatial justice integrates principles of social justice with geographical considerations and is broadly concerned with fairness in the spatial organization of public resources (13). In existing literature, spatial justice theory highlights that spatial inequality may arise not only from uneven physical distribution, but also from broader structural conditions that shape access opportunities across space, such as transportation systems, urban form, and institutional arrangements (14). The spatial agglomeration and marginalization of medical resources reflect differences in service accessibility for different social groups along the geographical dimension, thereby affecting the realization of their health rights (15). Spatial justice theory is concerned not only with the physical distribution of resources but also emphasizes whether the spatial organizational structure provides fair service opportunities for all residents. Even when the overall number of medical facilities increases, mismatches between facility locations and population concentrations may result in spatial “service blind spots.” Such spatial mismatches are commonly discussed in the spatial justice literature as potential manifestations of inequitable access, rather than as direct evidence of injustice per se (16). Within the existing urban development pattern, the uneven distribution of medical resources may undermine the policy goal of “universal access to basic medical care.” In this context, introducing a spatial justice framework helps move beyond a purely quantity-based evaluation of facilities and encourages attention to spatial relationships between resource distribution and population patterns, providing an interpretive basis for identifying potential equity concerns in medical facility layouts. Furthermore, the spatial configuration of medical resources is not only the result of health policies but is also influenced by multiple social environmental factors such as transportation accessibility, urban construction density, and economic development level (17). Therefore, assessments of medical resource equity should consider spatial layout in conjunction with broader urban environmental contexts, such as transportation accessibility, urban form, and development intensity, which jointly shape patterns of access.

Based on the above practical context and theoretical background, how to systematically identify the spatial distribution characteristics of medical resources, scientifically assess their equity level, and further analyze the influencing mechanisms of their spatial distribution have become key issues for achieving balanced regional medical allocation and enhancing residents’ medical well-being. This study selects four representative cities in Shandong Province—Qingdao, Jinan, Yantai, and Weihai, as the study area. Based on medical facility POI (Point of Interest) data provided by Amap, it systematically conducts an analysis of the spatial distribution characteristics and equity of medical resources. The research first applies spatial autocorrelation analysis methods, including Global Moran’s I and Local Indicators of Spatial Association (LISA), to quantify the spatial agglomeration characteristics of medical facilities in the four cities and reveal their spatial distribution patterns. Secondly, the Gini coefficient is used to measure the distributional equity of medical facilities, depicting the degree of balance in medical resource allocation within the region. Finally, using the Geographically Weighted Regression (GWR) model, and introducing variables such as building coverage rate, road network density, nighttime light value, population size, and regional area, the study explores the mechanisms through which these spatial and socio-economic factors influence the spatial distribution of medical resources.

Through the above multi-dimensional quantitative analysis, this study aims to deeply reveal the spatial pattern and equity characteristics of medical resource allocation in the four cities of Shandong Province, identify the core factors affecting resource distribution, and accordingly propose targeted policy recommendations to contribute to the optimization and balanced development of the regional medical service system. The research results can not only provide a reference basis for scientific decision-making by local governments in medical resource planning and layout but also serve as a reference for empirical research on medical resource equity in other regions, helping to improve the overall level of medical resource allocation in China and promote the goals of social equity and health equity.

## 2 Literature review

### 2.1 Review of methods for measuring medical resource equity

The measurement of medical resource equity is a core topic in public health and health geography, aiming to identify resource allocation disparities across space and population groups, thereby evaluating whether the distribution is reasonable and fair. In existing research, the most common measurement methods mainly fall into two categories: statistical indicator methods based on quantity distribution and geographical analysis methods based on spatial processes. The former, represented by statistical indicators such as the Gini coefficient and Atkinson index, can quantify the degree of distribution inequality of medical resources among regions and is suitable for equity evaluation at the macro scale (18). The Gini coefficient, due to its simple calculation and wide applicability, is widely used to measure the equity of allocation of the number of medical institutions, number of beds, or physician resources among administrative districts or grid units (19–21). Similar statistical approaches have been extensively applied in international contexts, such as evaluating the long-term inequality in the geographical distribution of general practitioners in England and Wales (22), and assessing the urban–rural disparities in healthcare infrastructure across European countries (23, 24).

The latter emphasizes incorporating the spatial dimension into equity evaluation, primarily using methods such as spatial autocorrelation analysis (e.g., Global Moran’s I) (25, 26), spatial accessibility measurement (e.g., the Two-Step Floating Catchment Area (2SFCA) method and its improvements) (27–29), and kernel density estimation (30–32). These methods can reveal the spatial coupling relationship between medical resources and population, identify issues of spatial agglomeration, resource blind spots, or structural absence, providing a basis for subsequent resource optimization and planning. Building upon this methodological foundation, our study contributes to the international discourse on spatial justice by utilizing the aforementioned metrics to assess the unique urban context of China. While similar spatial evaluations have been conducted in nations such as the United States (33), Japan (34), Italy (35), and Romania (36), research that addresses the specific complexities of China’s rapid urbanization and land-use structure remains essential. By applying these indicators, this study extends the global evidence base for healthcare accessibility to Shandong, China, and provides a transferable analytical framework for other regions facing comparable infrastructural constraints.

Overall, different measurement methods have their own advantages: statistical indicators are suitable for macro-level equilibrium evaluation, while spatial analysis methods emphasize geographical variability and service accessibility.

### 2.2 Analysis of spatial influencing factors of medical resources

The formation of the spatial distribution of medical resources is influenced by a combination of various natural, social, and institutional factors, exhibiting significant spatial heterogeneity. Existing research indicates that medical facilities are often preferentially located in areas with high population density, high urban hierarchy, and active economic activity (37, 38). This reflects the spatial coupling relationship between service demand scale, government investment capacity, and facility operational efficiency. Population is also a fundamental guiding variable for medical resource allocation (39). Population agglomerations are typically also areas with high demand for medical services, and locating medical facilities there can achieve economies of scale and service maximization. In terms of spatial form, urban form indicators such as building density and road network density affect the spatial location and service scope of medical resources (40, 41). For example, road network density directly relates to the convenience of residents seeking medical care and is an important reference factor for medical resource layout.

Although most existing studies have identified key influencing factors for the spatial distribution of medical resources, the commonly used global linear regression methods have obvious limitations: they assume that “the mechanism of influencing factors is constant in space,” which fails to capture regional structural differences and underestimates the spatial heterogeneity of policy responses.

### 2.3 Spatial justice in explaining medical resource imbalance

In recent years, “Spatial Justice” has gradually been introduced into medical resource equity research as a theoretical framework that is both normative and analytical (42, 43). Current mainstream research on the spatial equity of medical service facilities is mostly based on egalitarian theory. From the perspective of political philosophy, the core idea of egalitarianism emphasizes the equality of all people. Introducing this concept into the healthcare field, the core concern of spatial justice theory is whether medical services provide equal service opportunities for residents in different spatial units.

Rosenberg (44) criticized health geography’s long-term over-reliance on surface indicators such as “distance” and “service accessibility,” pointing out its lack of systematic exploration of justice theory itself when measuring social justice. An appropriate example is that even if two regions possess a comparable number of medical facilities, significant differences in their transportation connectivity, population structure, or social capital can still result in a huge gap in residents’ actual access to medical services. Rosenberg further pointed out that the spatial form of resource imbalance often stems from underlying institutional choices and spatial governance logic. It is necessary to proceed from a “non-idealist” perspective of justice to explore how to enhance relative fairness among regions through planning interventions under limited resources and complex realities. This proposition challenges the traditional “distribution balance” logic and calls for embedding social justice theory into health geography research to address complex socio-spatial inequality issues. Even though the government continuously increases the number of medical facilities, the lack of optimized spatial planning and weakened intervention in marginal areas leads to a large population being outside the “formal service area,” reflecting a typical problem of spatial justice imbalance. This suggests that when evaluating the spatial equity of medical resources, we should go beyond simple accessibility or aggregate analysis and pay more attention to the “potential inequality” mechanisms within the spatial structure.

In summary, spatial justice theory provides an important value judgment perspective and theoretical explanatory framework for the phenomenon of medical resource imbalance, helping to shift the focus from “resource distribution” to “social outcomes,” and providing theoretical support for building a more inclusive and equitable urban medical system.

## 3 Methodology

### 3.1 Study area and data

#### 3.1.1 Study area

This study selects Qingdao, Jinan, Yantai, and Weihai cities in Shandong Province as the study area. These four cities represent different types of economic development models and geographical characteristics within Shandong Province, providing strong representativeness.

(1) Qingdao: As the city with the largest economic output in Shandong Province, Qingdao is a nationally important coastal central city with abundant medical resources and high medical standards. Its medical facilities are mainly concentrated in core urban areas such as Shinan District and Shibei District, while suburban areas and some towns have relatively fewer medical resources.
(2) Jinan: As the provincial capital of Shandong, Jinan is the political, economic, and cultural center of the province. Medical resources in Jinan are relatively concentrated in central urban areas like Lixia District and Shizhong District, while uneven distribution exists in surrounding areas.
(3) Yantai: Yantai is an important coastal city in Shandong Province with a relatively high level of economic development. The distribution of medical resources is relatively balanced, but certain disparities in medical service levels still exist in some county areas, especially where primary medical infrastructure in towns is relatively weak.
(4) Weihai: Weihai is one of the economically developed cities in Shandong Province and also a nationally renowned livable city. Its medical resources are relatively concentrated in the main urban area, but accessibility to high-quality medical resources remains insufficient in some remote towns.

**Figure 1:**
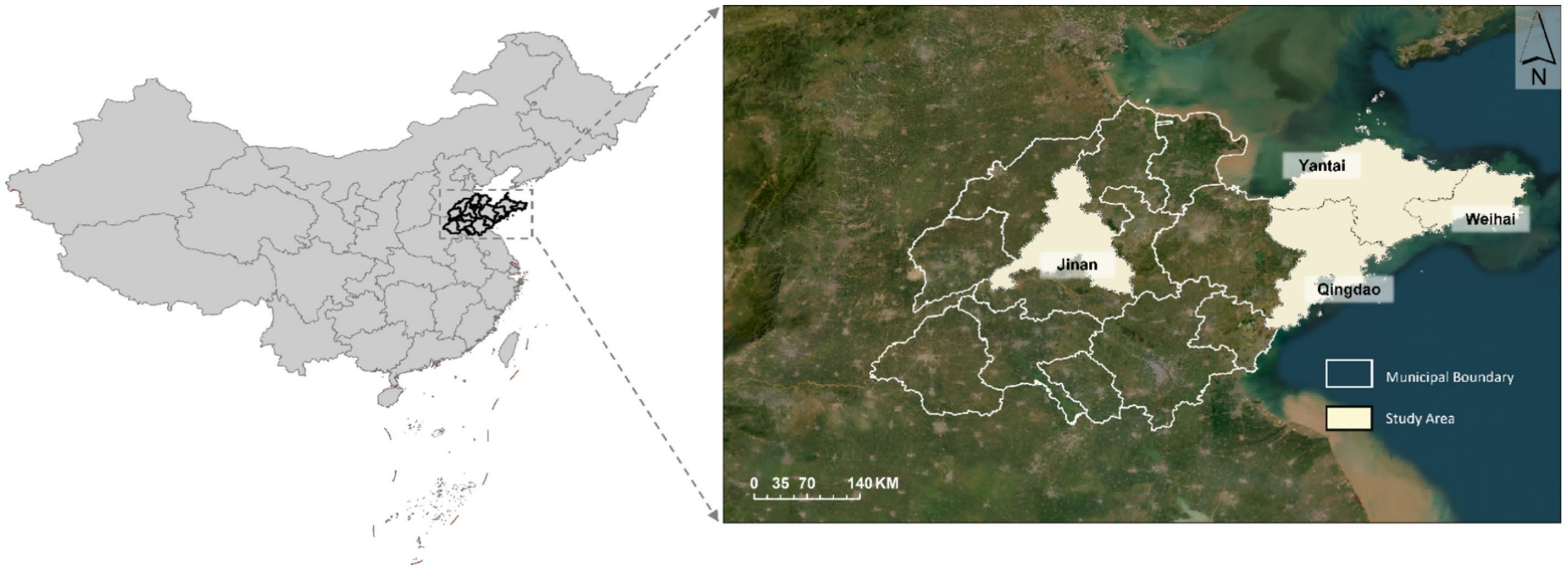
Medical study area map (Map approval number: GS(2024)0650). Base map data sourced from the ministry of natural resources of China.

The locations of the four selected cities in Shandong Province are shown in Figure 1. Selecting these four cities as the study area can comprehensively reflect the spatial distribution characteristics of medical resources under different economic development levels, population densities, and geographical environments in Shandong Province, and provide more targeted reference basis for optimizing medical resource allocation.

#### 3.1.2 Research data

The data sources for this study include medical facility data and influencing factor data. Influencing factor data include population data, road data, nighttime lights, and building footprint data. The data names and sources are shown in Table 1. To achieve spatial matching of the data, this study spatially aggregates medical facility data based on community-level administrative boundaries, calculates the distribution of medical resources using the community as the basic unit, and conducts spatial correlation analysis for various influencing factor data. Through this method, we can systematically quantify the spatial distribution pattern of medical resources and its influencing factors at the community scale, providing data support for subsequent spatial analysis and model construction.

**Table 1:** Data names and sources.

Data name	Source
POI Data	https://www.amap.com
Population Data	https://figshare.com/s/d9dd5f9bb1a7f4fd3734
Road Data	https://www.openstreetmap.org
Building Data	https://www.tianditu.gov.cn/
NPP-VIIRS Nighttime Light Data	https://eogdata.mines.edu/products/vnl/

1) Dependent Variable: Medical Facilities

**Figure 2:**
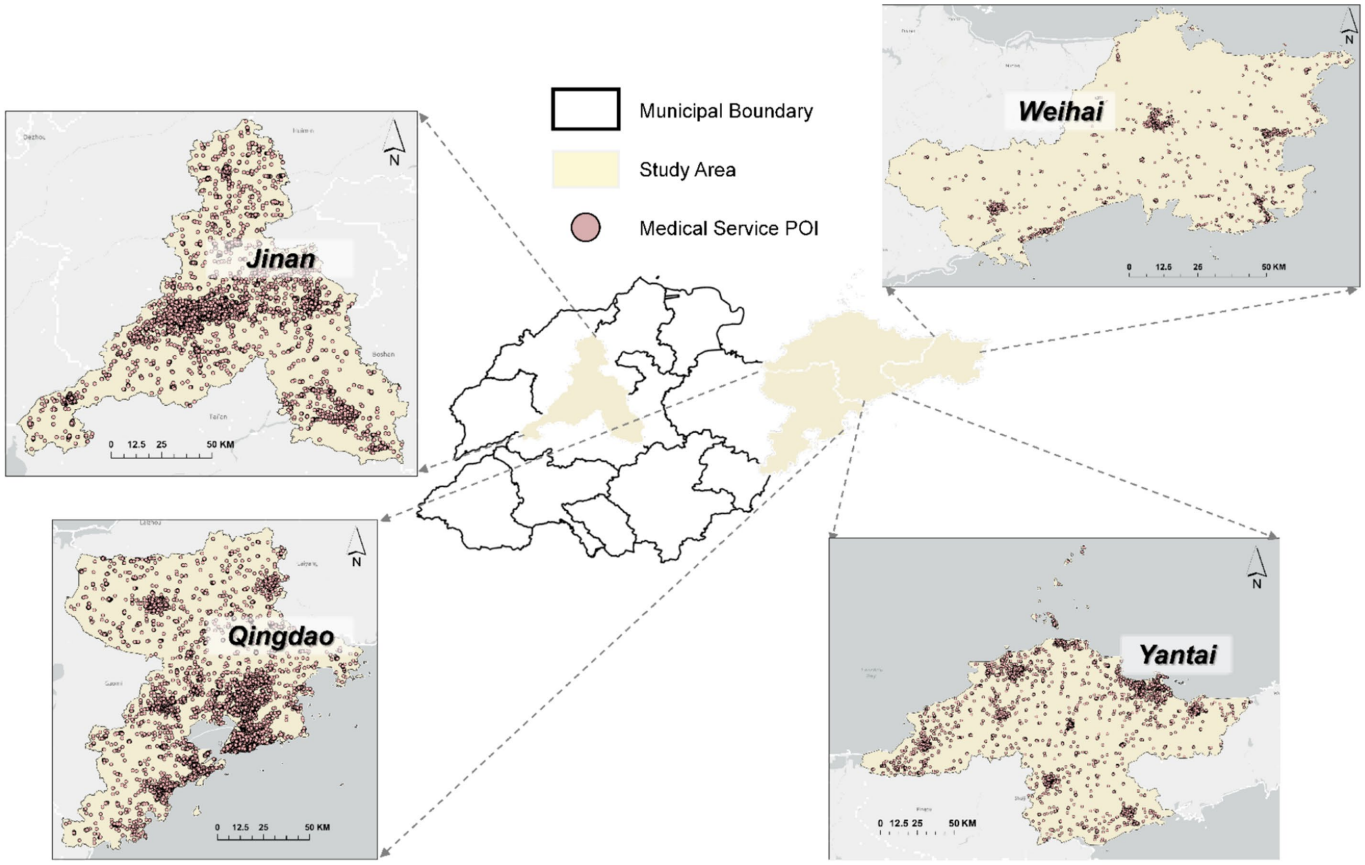
Medical service POI of the study area.

Medical facility data come from POI (Point of Interest) data provided by Amap, including various types of medical institutions such as general hospitals, specialized hospitals, community health service centers, and clinics. The spatial distribution of medical service POIs in the study area is visualized in Figure 2. This data can comprehensively reflect the spatial distribution of medical resources in the study area. We use the count of medical facility POIs as the dependent variable because it directly reflects the supply level of medical resources in different communities and exhibits high explanatory power in regression analysis. Furthermore, compared to medical facility density per unit area, the absolute POI count has more stable statistical properties in model fitting, avoiding bias caused by excessive differences in community area.

2) Influencing Factor Data

Many factors influence the spatial distribution of medical resources. This study primarily selects key variables such as population, transportation accessibility, and socio-economic level for analysis. Specific data sources are as follows:

1) Population Data

This study uses 100 m resolution population grid data from China’s Seventh National Population Census, developed by Professor Yuehong Chen’s team and shared on the Figshare platform. The team proposed a downscaling method based on Stacking Ensemble Learning and geospatial big data to process the seventh census data into high-precision 100-meter resolution population grid data, enhancing the refined expression of population spatial distribution. The high spatial resolution (100 m) of this data enables it to accurately characterize population distribution characteristics within cities and at urban–rural junctions, providing a solid data foundation for this study’s analysis of the match between medical resources and population.

2) Road Network Data

In areas with high road network density, residents can access required services more conveniently, potentially leading to higher satisfaction with public services. The road network data for Shandong Province used in this study was obtained from OpenStreetMap. To quantify the impact of the transportation network on the accessibility of medical resources, this study calculates road network density based on the road data. The calculation formula is as follows (Equation 1):


(1) $$\delta =\frac{\sum x}{S}$$

Where, ∑x is the total length of roads within the administrative area, and S is the area of the administrative area. A higher *δ* may indicate better traffic permeability; whereas a lower δ may indicate traffic congestion or poor permeability.

3) Nighttime Light Data

Nighttime light data, also known as nightlight remote sensing data, uses remote sensing technology to observe the Earth’s nighttime glow from space. Compared to ordinary remote sensing satellite images, nightlight remote sensing provides a unique perspective centered on human activities, directly revealing underlying patterns of surface human activity. The NPP VIIRS nighttime light data used in this study comes from the NCEI (National Centers for Environmental Information) under NOAA (National Oceanic and Atmospheric Administration).

Since nighttime light data can, to some extent, reflect the level of urbanization and resident income levels, this study uses it as an important factor influencing the spatial distribution of medical resources. By combining nighttime light data with the spatial distribution of medical facilities, this study aims to further reveal the potential relationship between urban development and medical resource equity, providing a scientific basis for optimizing medical service supply.

4) Building Data

This paper uses building density as a quantitative indicator, utilizing building vector data for the study area from Tianditu. Building density is an important indicator for measuring spatial development intensity and urban functional layout. A higher value usually implies greater concentration of population and economic activities, and consequently higher demand for medical resources. Theoretically, areas with higher building density often have higher population density and more active socio-economic activities, and medical institutions tend to agglomerate in these areas to meet residents’ medical needs.

Building density refers to the ratio of the building projection area to the administrative area, reflecting the vacant land rate and building concentration degree within the administrative area. The calculation formula is as follows (Equation 2):


(2) $$D=\frac{\sum_{i=1}^{n}A_{i}}{S}$$

Where, $A_{i}$ represents the building coverage rate of the administrative area i , n is the total number of buildings, D represents the projected area of the building, and S is the area of the administrative area.

### 3.2 Spatial agglomeration calculation

This study uses Global Moran’s I to assess the overall spatial autocorrelation of medical resources and further employs Local Indicators of Spatial Association (LISA) to identify spatial distribution patterns of medical facilities within different regions.

#### 3.2.1 Global spatial autocorrelation analysis (global Moran’s I)

Moran’s I is a classic measure of spatial autocorrelation used to quantify the degree of clustering or dispersion of a variable in space. Its calculation formula is as follows (Anselin) (Equation 3):


(3) $$I=\frac{n}{\sum i\sum jW_{\mathrm{ij}}}\times \frac{\sum i\sum jW_{\mathrm{ij}}}{\sum i}\frac{\left(Y_{i}-\bar{Y})(Y_{j}-\bar{Y}\right)}{{\left(Y_{i}-\bar{Y}\right)}^{2}}$$

Where, n represents the total number of communities; $Y_{i}$ and $Y_{j}$ represent the number of medical facilities in community i and community j , respectively; $\bar{Y}$ is the mean number of medical facilities across all communities; $W_{\mathrm{ij}}$ is the spatial weight between community i and community j . I is the sum of all spatial weights. The value range of Moran’s I is [−1, 1], which can indicate three types of autocorrelation:

Moran’s I > 0 indicates positive spatial autocorrelation, meaning medical facilities exhibit a clustered pattern (high-high or low-low adjacency).

Moran’s I < 0 indicates negative spatial autocorrelation, meaning medical facilities exhibit a dispersed pattern (high-low or low-high adjacency).

Moran’s I = 0 or close to 0 indicates no spatial autocorrelation, meaning medical resources are randomly distributed.

#### 3.2.2 Local spatial autocorrelation analysis (LISA)

The Global Moran’s I can only reveal the overall trend and cannot identify which specific areas form spatial clusters or dispersion patterns. Therefore, we further use Local Indicators of Spatial Association (LISA) to assess the spatial clustering of medical facilities within each community. The LISA index calculation formula is as follows (Anselin) (Equation 4):


(4) $$I_{i}=(Y_{i}-\bar{Y})\sum_{j}W_{\mathrm{ij}}(Y_{j}-\bar{Y})$$

The LISA index can be used to classify spatial cluster patterns, including:

High-High cluster (HH): A high-medical-resource community surrounded by neighboring high-resource areas (medical facility enrichment area).

Low-Low cluster (LL): A low-medical-resource community surrounded by neighboring low-resource areas (medical resource shortage area).

High-Low cluster (HL): A high-medical-resource community surrounded by low-resource areas, potentially indicating an area of resource imbalance.

Low-High cluster (LH): A low-medical-resource community surrounded by high-resource communities, possibly reflecting the spillover effects of medical resources.

### 3.3 Spatial equity calculation

Spatial equity is an important indicator for measuring the balance of resource distribution, especially in medical resource allocation research. The Gini coefficient is widely used to assess the equity of medical facility distribution among different regions. The Gini coefficient originates from the field of economics, used to measure the inequality of income or wealth distribution, and has later been extended to multiple research fields including healthcare, education, and public resource allocation. This study uses the Gini coefficient to quantify the spatial equity of medical facilities at the community level and explores its spatial pattern combined with spatial autocorrelation analysis. The Gini coefficient is an inequality measurement method based on the Lorenz Curve, with values between 0 and 1: G = 0 indicates completely equal resource distribution, meaning all areas have the same number of medical facilities; G = 1 indicates complete inequality, meaning all medical facilities are concentrated in a single area, with no medical resources in the remaining areas. The Gini coefficient is calculated based on the following formula (Equation 5):


(5) $$G=1-\sum_{i=1}^{n}(P_{i}-P_{i-1})(Q_{i}+Q_{i-1})$$

Where, $P_{i}$ is the cumulative proportion of communities; $Q_{i}$ is the cumulative proportion of medical facilities. This method is only suitable for overall equity analysis and cannot reflect local spatial differences. As a supplement, this study also introduces a spatial weight matrix and proposes a local Gini index calculated for spatial equity between the community and its neighbors, its formula is as follows (Equation 6):


(6) $$G_{i}=\frac{\sum_{i=1}n_{i}\sum_{j=1}n_{j}W_{\mathrm{ij}}|Y_{i}-Y_{j}|}{2n_{i}\sum_{j=1}n_{j}Y_{j}}$$

Where $n_{i}$ is the number of spatial units in the neighborhood set of community i, as determined by the spatial weight matrix Wij.

### 3.4 Geographically weighted regression model

This paper uses the GWR model, the model is formulated as Equation 7. This model studies spatial heterogeneity by assigning coefficient values based on the varying degrees of influence of independent variables on the dependent variable. Therefore, the GWR model can provide more refined and accurate results when analyzing the effects of various influencing factors on the number of medical resources in different regions.


(7) $$Y_{i}=\beta_{0}(u_{i},u_{j})+\sum_{k=1}^{k}\beta_{k}(u_{i},u_{j})x_{\mathrm{ik}}+\theta_{i}$$

Where, $Y_{i}$ is the dependent variable, i.e., the number of medical facilities; $\;(u_{i},u_{i})$ represents the central point coordinates of the i -th community; $\;\beta_{k}(u_{i},v_{i})\;$ represents the regression coefficient for variable k in the i -th community; $x_{\mathrm{ik}}$ is the attribute value of indicator variable k in that spatial grid; $\theta_{i}$ represents the random error.

This study applies the GWR model to examine spatial heterogeneity in the relationships between medical facility distribution and its influencing factors. Unlike global regression models, GWR allows regression coefficients to vary across space by incorporating a spatial weight matrix that reflects local neighborhood structures. In this study, the spatial weight matrix Wij is constructed using Queen contiguity, ensuring appropriate neighborhood definitions for irregular spatial units. Furthermore, an adaptive bandwidth is selected through AICc-based optimization using a golden section search algorithm, enabling robust local parameter estimation across heterogeneous urban contexts.

## 4 Results

### 4.1 Analysis of medical spatial pattern

**Figure 3:**
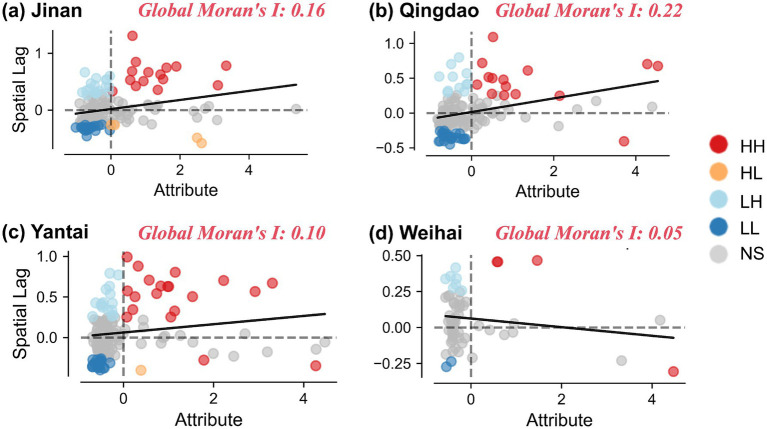
Moran’s *I* and LISA calculation results for Jinan **(a)**, Qingdao **(b)**, Yantai **(c)**, and Weihai **(d)**. Moran scatter plots (left) display the global Moran’s *I* values; LISA cluster maps (right) identify high-high (HH), low-low (LL), high-low (HL), and low-high (LH) spatial clusters of medical facilities.

The spatial autocorrelation characteristics of medical resources in the four cities were assessed using Global Moran’s I and Local Indicators of Spatial Association (LISA). The results are visualized in Figure 3 (Moran’s I and LISA calculation).

Jinan: The scatter plot shows most points distributed in the positive area (red indicates high-value areas, blue indicates low-value areas). The Global Moran’s I is 0.16, indicating a mild positive spatial autocorrelation in Jinan. The plot shows some HH and LL cluster points, indicating that in certain areas of Jinan, areas with higher or lower attribute values exhibit spatial clustering.

Qingdao: The Global Moran’s I is 0.22, slightly higher than Jinan, indicating slightly stronger spatial autocorrelation in Qingdao. The scatter plot shows that high-value (HH) and low-value (LL) areas still exhibit obvious clustering, possibly due to certain regional differences in Qingdao’s urban development model causing spatial clustering of attribute values.

Yantai: The Global Moran’s I is 0.10, close to 0, indicating weak spatial autocorrelation in Yantai, with attribute values distributed relatively randomly in space. The scatter plot shows some HL and LH points, indicating that the oppositional relationship between high-value and low-value areas is relatively obvious in Yantai, but the overall spatial distribution shows no significant clustering trend.

Weihai: The Global Moran’s I is 0.05, similar to Yantai, indicating weak spatial autocorrelation in Weihai, with relatively uniform distribution of attribute values. The scatter plot shows that the points in Weihai are quite dispersed, with almost no obvious clustering areas, and most points are located in low-value (LL) or high-value (HH) areas.

### 4.2 Equity calculation results

**Figure 4:**
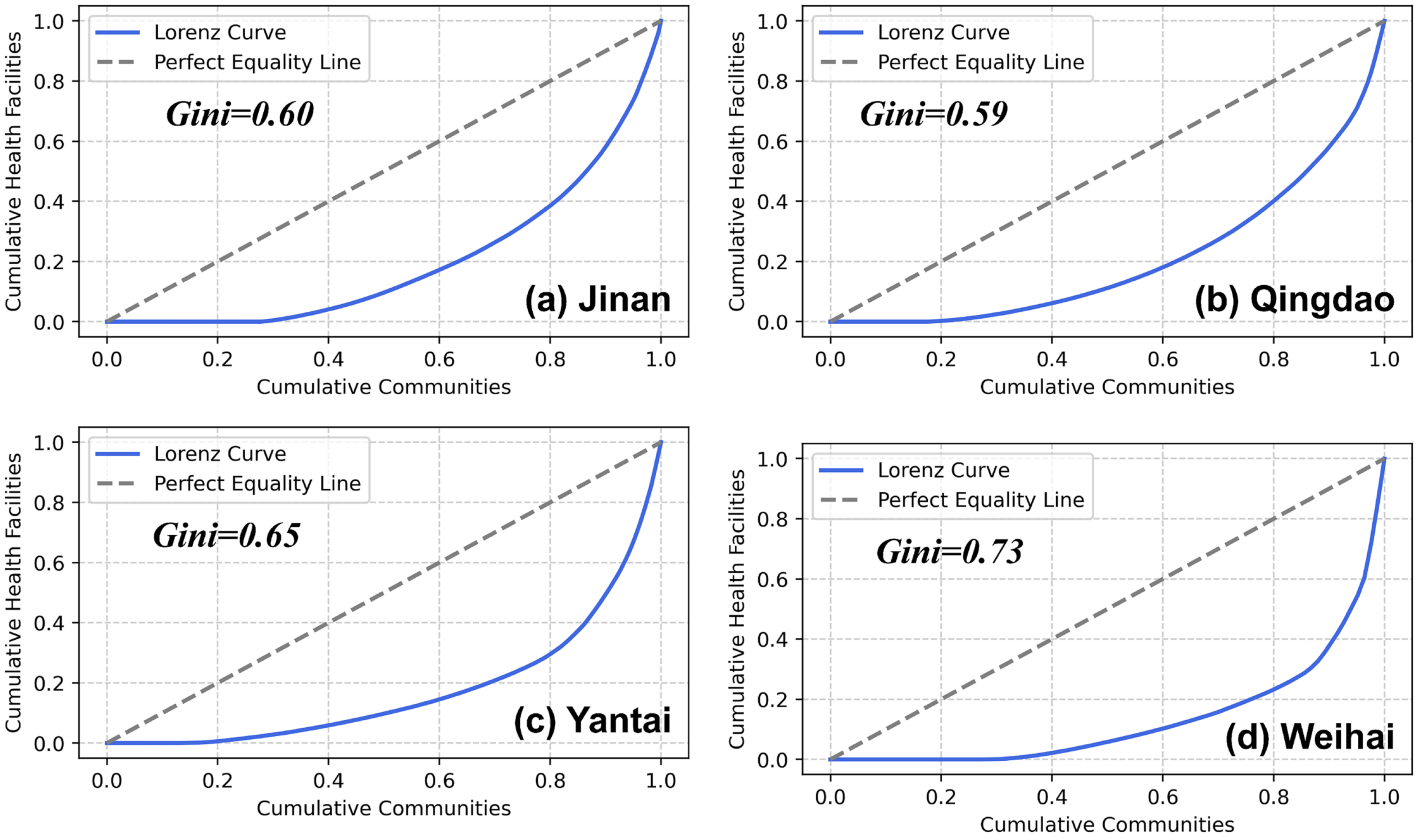
Lorenz curve and Gini coefficient results for Jinan **(a)**, Qingdao **(b)**, Yantai **(c)**, and Weihai **(d)**.

This study calculated the Gini coefficient for medical facilities within the study area and plotted the Lorenz curve in Figure 4. When the Gini coefficient is close to 0, it indicates a relatively balanced distribution of medical resources, with communities enjoying relatively equitable medical services. When the Gini coefficient is close to 1, it means medical facilities are highly concentrated in a few communities, indicating obvious imbalance in resource configuration. According to the calculation results, Qingdao (0.59) and Jinan (0.60) have relatively low Gini coefficients, indicating a more balanced distribution of medical facilities in these two cities. In contrast, Yantai (0.65) and Weihai (0.73) have higher Gini coefficients, indicating more prominent imbalance in medical resource distribution in these two cities. Particularly, Weihai has the highest Gini coefficient, suggesting that medical facilities in this city are more concentrated in a few communities, while some areas may suffer from insufficient medical resources.

Overall, there is a certain degree of imbalance in the distribution of medical resources within the study area, and the level of equity varies among cities. Possible influencing factors include population density, economic development level, transportation accessibility, and government medical investment. Cities with relatively fair medical resource distribution (such as Qingdao and Jinan) usually have a more complete medical system and relatively balanced infrastructure, while cities with imbalanced resource distribution (such as Weihai and Yantai) may be influenced by geographical factors and urbanization levels, leading to the concentration of medical facilities in central urban areas and relative scarcity in peripheral areas.

**Figure 5:**
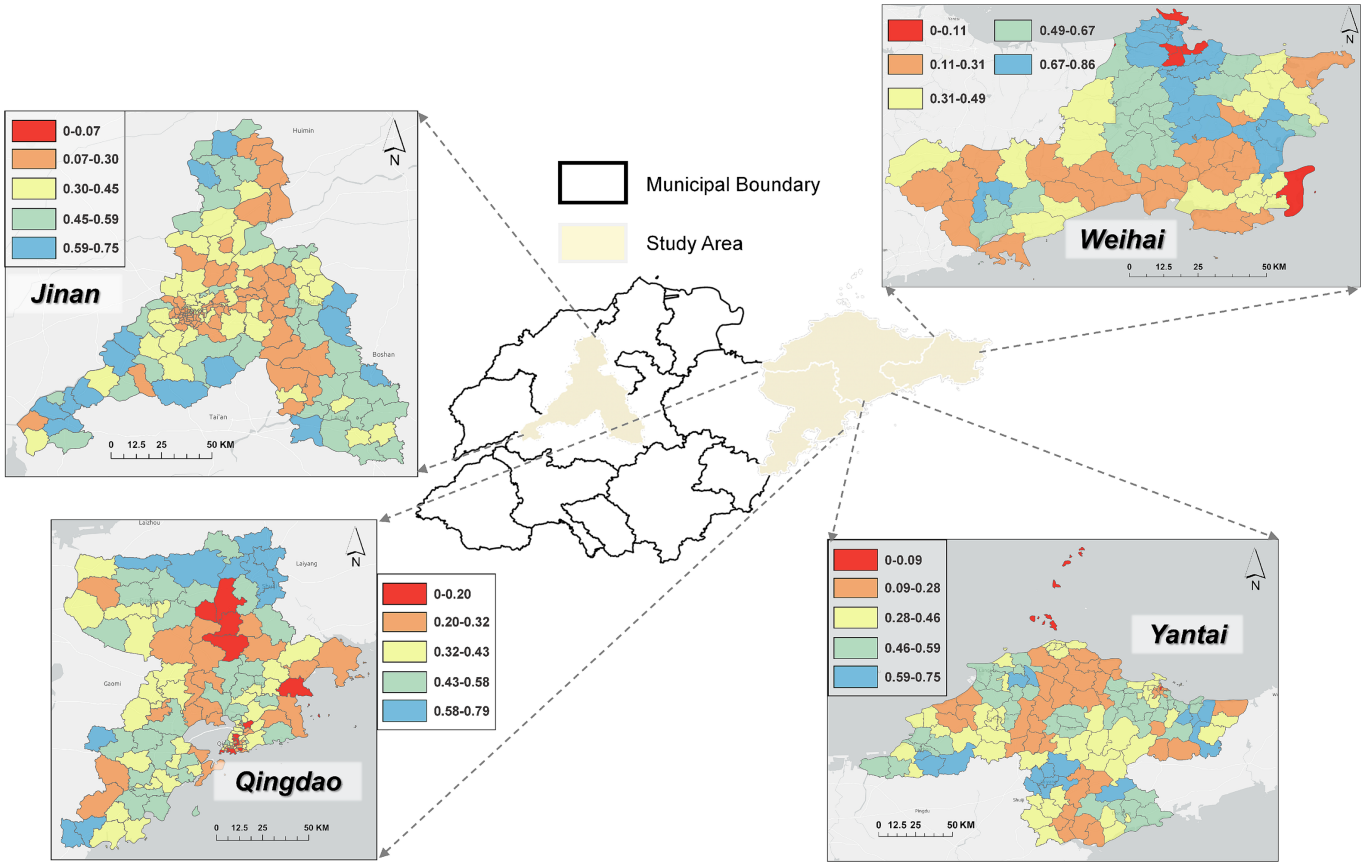
Local Gini coefficient maps for Jinan, Qingdao, Yantai, and Weihai. Values are classified using the natural breaks method; higher values indicate greater inequity in medical facility distribution within the local neighborhood.

Based on the global Gini coefficient, this study further calculated the Local Gini Coefficient to reveal differences in the equity of medical facility distribution across different regions in Figure 5. The Natural Breaks method was used to classify the local Gini coefficients. A higher value indicates more unfair distribution of medical facilities in that region and its adjacent areas. Spatially, high Gini areas (unfair areas) in all cities are mainly concentrated on the urban fringes. In Jinan, the local Gini coefficient shows a gradual gradient increase from the city center toward the edges. In Qingdao, the equitable areas are relatively concentrated. Apart from the high equity in Shinan and Shibei Districts, Licang District, Jimo District, Laixi City, and Pingdu City also show relatively balanced medical resource distribution. In contrast, equitable areas in Weihai are sporadically distributed without forming obvious spatial clusters, indicating a relatively discrete spatial configuration of medical resources. In Yantai, the most equitable areas surprisingly appear in island regions like Cheyou Island and the Miaodao Archipelago, which might be related to the lower demand for medical resources in these areas.

**Figure 6:**
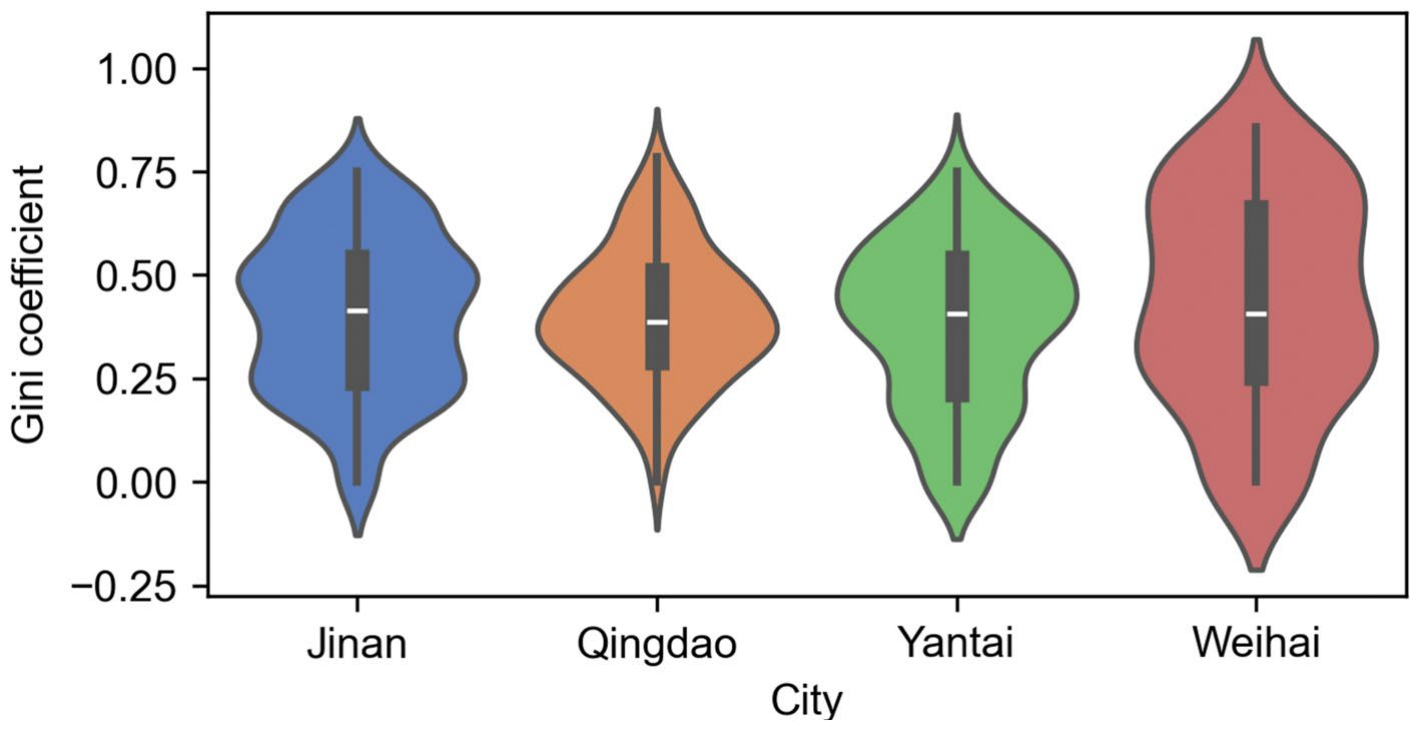
Violin plot of local Gini coefficients for each city. The width of each violin represents the density of community-level local Gini values; the median and interquartile range (IQR) are shown within.

Further analyzing the distribution characteristics of the local Gini coefficient combined with the violin plot in Figure 6, it can be found that the violin plots for Qingdao and Jinan are relatively symmetrical and concentrated, indicating that the local Gini coefficient varies little among most communities in these two cities, and the medical resource allocation is relatively stable. Among them, Qingdao has a lower median and a narrower IQR (Interquartile Range), indicating higher equity of medical facilities; while Jinan has a slightly wider IQR, indicating some imbalance still exists in some communities.

The violin plot for Yantai is slightly skewed with a wide IQR, indicating greater variability in the local Gini coefficient. This means there are large disparities in medical resource allocation between different areas in Yantai, possibly caused by the uneven distribution of medical facilities between urban areas and rural areas. Medical resources are more concentrated in some urban areas, while medical facilities are scarce in some communities.

The violin plot for Weihai is the widest, has the largest distribution range, and shows a clear long-tail phenomenon (upper tail stretched), indicating the greatest fluctuation in medical facility equity in this city. Its local Gini coefficient is generally high, and the median is significantly higher than other cities, indicating that medical facilities in Weihai are highly concentrated in a few communities, with extremely uneven resource configuration, which may prevent residents in some areas from accessing medical services easily.

In summary, this study finds that Jinan and Qingdao have relatively high spatial equity of medical resources, Yantai exhibits certain urban–rural disparities in medical resource distribution, while Weihai has the most concentrated medical resources and the worst equity. To address the issue of uneven medical resource distribution, it is recommended to strengthen support for urban fringe areas, urban–rural junctions, and medically underserved areas in future medical resource allocation to enhance overall medical equity and accessibility.

### 4.3 GWR model results

#### 4.3.1 Overall results

To further explore the spatial influencing factors of medical facility distribution, we first conducted a global Ordinary Least Squares (OLS) regression as a baseline. The diagnostic tests revealed significant spatial non-stationarity across all four cities, with the Moran’s I of OLS residuals being consistently greater than 0 (*p* < 0.01). This indicated that a global model could not adequately capture the spatial dependencies in the data. Subsequently, to further explore the spatial influencing factors of medical facility distribution, this study constructed a Geographically Weighted Regression (GWR) model, using Building Coverage Rate (BCR), Road network Density (RD), Nighttime Light Value (VNL), Population (Pop), and Regional Area (Area) as independent variables to analyze the impact of these factors on medical facility distribution. The GWR model demonstrated a substantial improvement in explanatory power over the OLS model, as evidenced by a consistently higher R^2^ across all study areas. Table 2 shows the average regression coefficients and the coefficient of determination (R^2^) for the GWR model in each city.

**Table 2:** GWR average coefficients and R-squared table.

City	BCR	RD	VNL	Pop	Area	R^2^
Jinan	−0.009	0.072	0.16	0.897	0.031	0.932
Qingdao	−0.105	0.092	0.078	1.134	−0.011	0.869
Yantai	−0.038	0.224	0.157	0.823	0.027	0.801
Weihai	−0.11	0.577	−0.169	0.892	0.052	0.929

Judging from the coefficient of determination (R^2^), the GWR model has the best fit in Jinan (R^2^ = 0.932) and Weihai (R^2^ = 0.929), indicating that the model explains the spatial distribution of medical facilities well. The fit is relatively lower in Qingdao (R^2^ = 0.869) and Yantai (R^2^ = 0.801), suggesting that the model’s explanatory power is slightly weaker in these cities, and there may be other factors not included in the model that affect the spatial distribution of medical facilities.

(1) Building Coverage Rate (BCR)

The regression coefficient for Building Coverage Rate (BCR) is negative in all cities, indicating that areas with higher building density actually have fewer medical facilities spatially distributed. This may be due to intense land use competition in high-density building areas, limiting the siting of medical facilities.

Among them, Weihai (−0.110) and Qingdao (−0.105) are most affected, suggesting that medical resources in these two cities may be more concentrated in low-density areas rather than the building-dense core urban areas.

(2) Road Network Density (RD)

Road network density shows a strong positive influence on the spatial distribution of medical facilities, especially in Weihai (0.577) and Yantai (0.224), indicating that medical facilities in these cities tend to be located in areas with dense roads and convenient transportation.

The influence of road network density is relatively weaker in Qingdao (0.092) and Jinan (0.072), possibly because the overall road network in these two cities is well-developed, and medical facility siting does not rely entirely on road network density.

(3) Nighttime Light Value (VNL)

Nighttime light value typically reflects economic activity and urban development level. In Jinan (0.160) and Yantai (0.157), VNL has a significant positive impact on medical facility distribution, indicating that medical resources in these cities are more concentrated in economically prosperous and populous areas.

The VNL coefficient for Weihai is negative (−0.169), indicating that its medical facilities may not be entirely concentrated in economically developed areas, but also have some distribution in areas with low VNL values. This might be related to the large urban–rural disparity in medical resources in Weihai.

(4) Population (Pop)

Population density is the most important factor influencing the distribution of medical facilities. The population regression coefficients for all cities are positive and relatively large, indicating that areas with larger population sizes have more medical facilities.

Qingdao (1.134) is most influenced by population, suggesting that the city’s medical resources are highly dependent on population distribution, and medical facility siting may primarily revolve around densely populated areas. The coefficients for Jinan (0.897), Weihai (0.892), and Yantai (0.823) are also high, further proving that population is a key driving factor in medical facility layout.

(5) Regional Area (Area)

The influence of regional area varies by city. The regression coefficients for Jinan (0.031), Yantai (0.027), and Weihai (0.052) are positive, indicating that larger areas have relatively more medical facilities, possibly because these cities have established more medical institutions in urban–rural fringes or suburban areas.

The regional area coefficient for Qingdao is negative (−0.011), possibly indicating that medical facilities in this city are more concentrated in small, high-population-density areas, while larger areas have fewer medical resources.

#### 4.3.2 Analysis of Jinan results

(1) Building Coverage Rate (BCR)

In the regression analysis for Jinan, BCR has the largest positive impact in Zhangqiu District, indicating that areas with higher building coverage have a relatively higher number of medical facilities. This phenomenon may be closely related to the rapid development of Zhangqiu District in the process of urbanization. As building density increases, infrastructure construction in the region, especially medical facilities, has been correspondingly improved and enhanced. However, BCR also shows a positive relationship in some other areas, such as Laiwu District and Gangcheng District, indicating synchronized growth between building density and medical resources. Nevertheless, in most areas, especially the urban core, BCR shows a negative correlation with medical facilities, meaning that areas with increasing building coverage actually see a decrease in medical facilities. This might be due to over-urbanization or excessive construction density in some areas, leading to unbalanced allocation of spatial resources, where the construction of medical facilities fails to keep pace with building growth.

(2) Road Network Density (RD)

RD shows a strong positive relationship in Laiwu District and Gangcheng District, indicating that increased road network density directly promotes the growth of medical facilities. This phenomenon reflects the promoting effect of a good transportation network on the layout of regional medical resources. Higher road network density not only improves residents’ travel convenience but also enhances the accessibility of medical facilities, promoting the construction and improvement of related facilities. However, in other areas, especially the downtown area, road network density shows a negative correlation with the distribution of medical facilities. This might be because in some high-density areas, the transportation network is too dense, leading to traffic congestion, which affects the effective allocation of medical resources.

(3) Nighttime Light Value (VNL)

In the northern and central parts of Jinan, VNL shows a significant positive impact, indicating that areas with higher nighttime light intensity tend to have more medical facilities. This phenomenon may be related to the activity level, economic development, and social activities in the city center. Particularly in the central Lixia District, as the core commercial area of Jinan, nighttime lights are brighter, and medical facilities are relatively abundant. This indicates that in these areas, high economic activity and population density drive the agglomeration and development of medical facilities.

(4) Population Density (Pop)

Population density has a significant positive impact on the central part of Jinan, especially Lixia District, indicating that more densely populated areas have a greater number of medical facilities. The central area of Jinan, as the main commercial and residential zone, has a highly concentrated population, which in turn generates increased demand for medical services. The development of medical facilities in these areas is highly correlated with population growth, reflecting the close link between demand and supply. In contrast, the impact of population in other areas is more uniform, but in sparsely populated areas, the distribution of medical facilities is more scattered and may be constrained by other factors.

(5) Regional Area (Area)

Regional area has a weak positive impact on most areas, indicating that larger areas generally have more medical facilities. Especially in the peripheral areas of Jinan, large urban–rural fringe areas are often key targets for medical facility layout. However, only in some central areas does the relationship between area and medical facilities show a stronger positive correlation, possibly because population and economic activities are more concentrated in these areas, promoting the concentrated layout of medical facilities.

**Figure 7:**
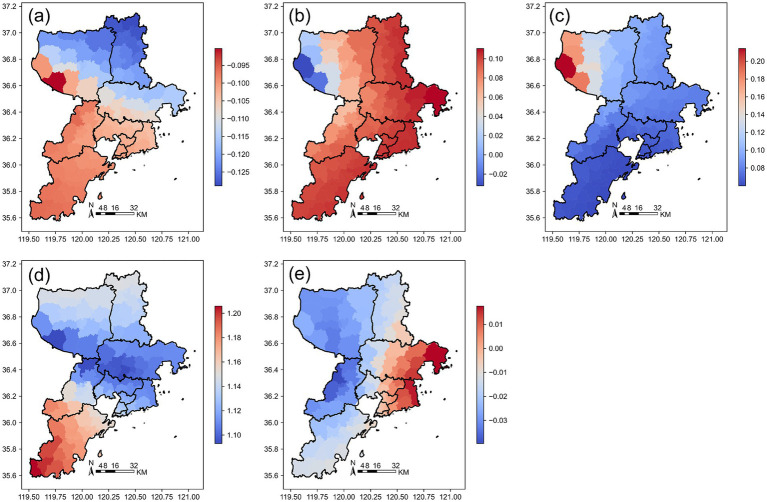
Spatial distribution of GWR regression coefficients in Jinan. **(a)** BCR; **(b)** RD; **(c)** VNL; **(d)** Pop; **(e)** Area. The color gradients indicate the relative magnitude of GWR coefficients, with red colors representing higher coefficient values and blue colors representing lower values. Positive coefficients indicate that the corresponding variable is positively associated with medical facility density at that location, while negative coefficients indicate an inverse association. Larger absolute values denote stronger local effects.

The spatial heterogeneity of these relationships across Jinan is further illustrated by the Geographically Weighted Regression (GWR) results. Figure 7 presents the spatial distribution of GWR regression coefficients in Jinan, showing coefficients for (a) Building Coverage Ratio (BCR), (b) Road Density (RD), (c) Nighttime Light Intensity (VNL), (d) Population (Pop), and (e) Area.

Different regions in Jinan exhibit significant spatial heterogeneity driven by factors such as building density, road network density, nighttime lights, population, and regional area. These factors influence the distribution and development of medical facilities through different mechanisms, presenting complex spatial patterns. In the urban core, increased building coverage and population density are often accompanied by the expansion of medical facilities, while in some peripheral areas, over-construction or dense transportation networks may lead to insufficient medical facilities.

#### 4.3.3 Analysis of Qingdao results

In Qingdao, the influence of each variable presents a relatively regular spatial distribution, overall showing obvious gradient changes. The specific analysis is as follows:

(1) BCR (Building Coverage Rate)

In Qingdao, the impact of BCR on medical facilities is negative, and its influence gradually increases from southwest to northeast. Although there are some differences in this negative relationship between different regions, the overall difference is not large and the influence is relatively weak. This indicates that areas with higher building coverage tend to have relatively fewer medical facilities, possibly related to over-urbanization and unbalanced resource allocation.

(2) RD (Road Network Density)

Road network density has a positive impact on most areas of Qingdao, showing that increased road network density is usually accompanied by an increase in medical facilities. However, the western part of Pingdu City shows a slight negative impact, indicating that increased road network density in this area may not have effectively promoted the construction of medical facilities, possibly due to low traffic flow or the transportation network failing to effectively connect important medical resource points.

(3) VNL (Nighttime Light Value)

The spatial distribution of VNL shows a trend opposite to that of road network density. In the western part of Pingdu City, nighttime lights have a strong positive impact on medical facilities, possibly closely related to economic activities and nighttime social activities in this area. In most other areas, the impact of nighttime lights on medical facilities is relatively weak and positive, indicating a loose relationship between nighttime light intensity and medical facilities, with limited influence.

(4) Population (Pop)

Population density has the greatest impact on medical facilities in Qingdao and is always positive. Especially in Huangdao District, the high population density most significantly drives the demand for medical facilities. This indicates that in more densely populated areas, particularly the rapid development of Huangdao District, promotes the concentrated construction and improvement of medical facilities, further reflecting the close connection between population and medical services.

(5) Regional Area (Area)

Regional area has a significant impact on the eastern parts of Qingdao (such as Jimo District, Laoshan District), showing a strong positive relationship, indicating that as the area increases in these regions, the distribution of medical facilities also gradually increases. However, in most other parts of Qingdao, the relationship between regional area and medical facilities is negative, meaning that larger areas may suffer from insufficient medical facilities due to dispersed resource allocation.

**Figure 8:**
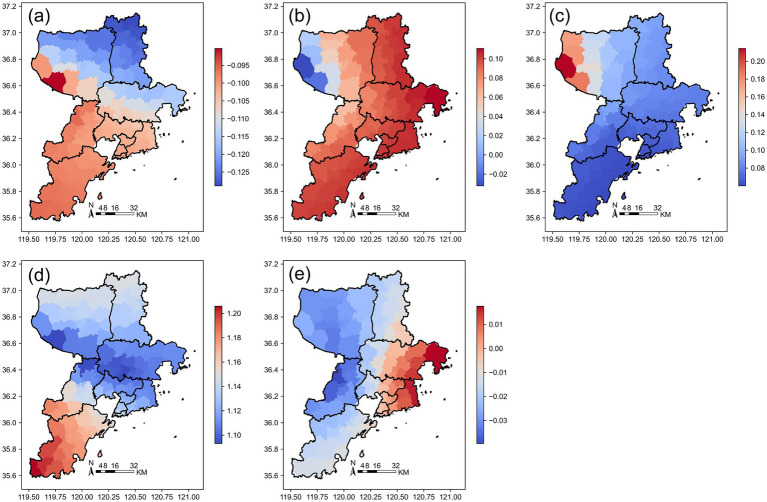
Spatial distribution of GWR regression coefficients in Qingdao. **(a)** BCR; **(b)** RD; **(c)** VNL; **(d)** Pop; **(e)** Area. The color gradients indicate the relative magnitude of GWR coefficients, with red colors representing higher coefficient values and blue colors representing lower values. Positive coefficients indicate that the corresponding variable is positively associated with medical facility density at that location, while negative coefficients indicate an inverse association. Larger absolute values denote stronger local effects.

The influence of explanatory variables exhibits pronounced spatial heterogeneity across Qingdao, with clear regional gradients in their effects. Figure 8 displays the spatial distribution of GWR regression coefficients in Qingdao, showing coefficients for (a) Building Coverage Ratio (BCR), (b) Road Density (RD), (c) Nighttime Light Intensity (VNL), (d) Population (Pop), and (e) Area.

Specifically, building coverage rate, road network density, and population density show stronger positive associations with medical facility distribution in the urban core and along the coast, reflecting the concentration of development and demand in these zones. Meanwhile, nighttime lights and regional area display more complex and spatially varied patterns, with localized hotspots of positive or negative influence. By analyzing this spatial heterogeneity, we gain deeper insight into the multifaceted drivers shaping medical resource allocation in Qingdao, offering a robust theoretical foundation for evidence-based urban planning and equitable healthcare infrastructure development.

#### 4.3.4 Analysis of Yantai results

In Yantai, the spatial influence patterns of various variables show certain regularity, but the differences in influence between different regions are relatively obvious. The specific analysis is as follows:

(1) BCR (Building Coverage Rate), Population (Pop), and Regional Area (Area)

The spatial influence patterns of these three variables show similar trends in Yantai, all having the greatest positive impact in the western areas of Laizhou District, Zhaoyuan District, and Longkou City. Increased building density, population density, and regional area in these areas all promote the construction and distribution of medical facilities. This might be because during the urbanization process in these regions, accompanied by population growth and increased building density, the demand for medical facilities rises accordingly, thus driving the expansion of medical resources.

(2) RD (Road Network Density)

Contrary to the influence of BCR, Pop, and Area, RD shows a positive impact in the eastern part of Yantai, and the impact is greatest there. This indicates that the denser road network system in the eastern region promotes the distribution of medical facilities, improves residents’ transportation accessibility, and drives the effective allocation of medical resources. The higher road network density and improved transportation network in the eastern area significantly enhance the accessibility and distribution density of medical facilities.

(3) VNL (Nighttime Light Value)

The impact of VNL on medical facilities in Yantai shows relatively localized spatial heterogeneity, with a strong positive relationship only in Laizhou District, indicating that areas with higher nighttime light intensity are usually accompanied by more medical facilities. This might be closely related to the economic activity level, nighttime social activities, and urbanization process in this region. In other areas, the impact of VNL on medical facilities is relatively weak.

**Figure 9:**
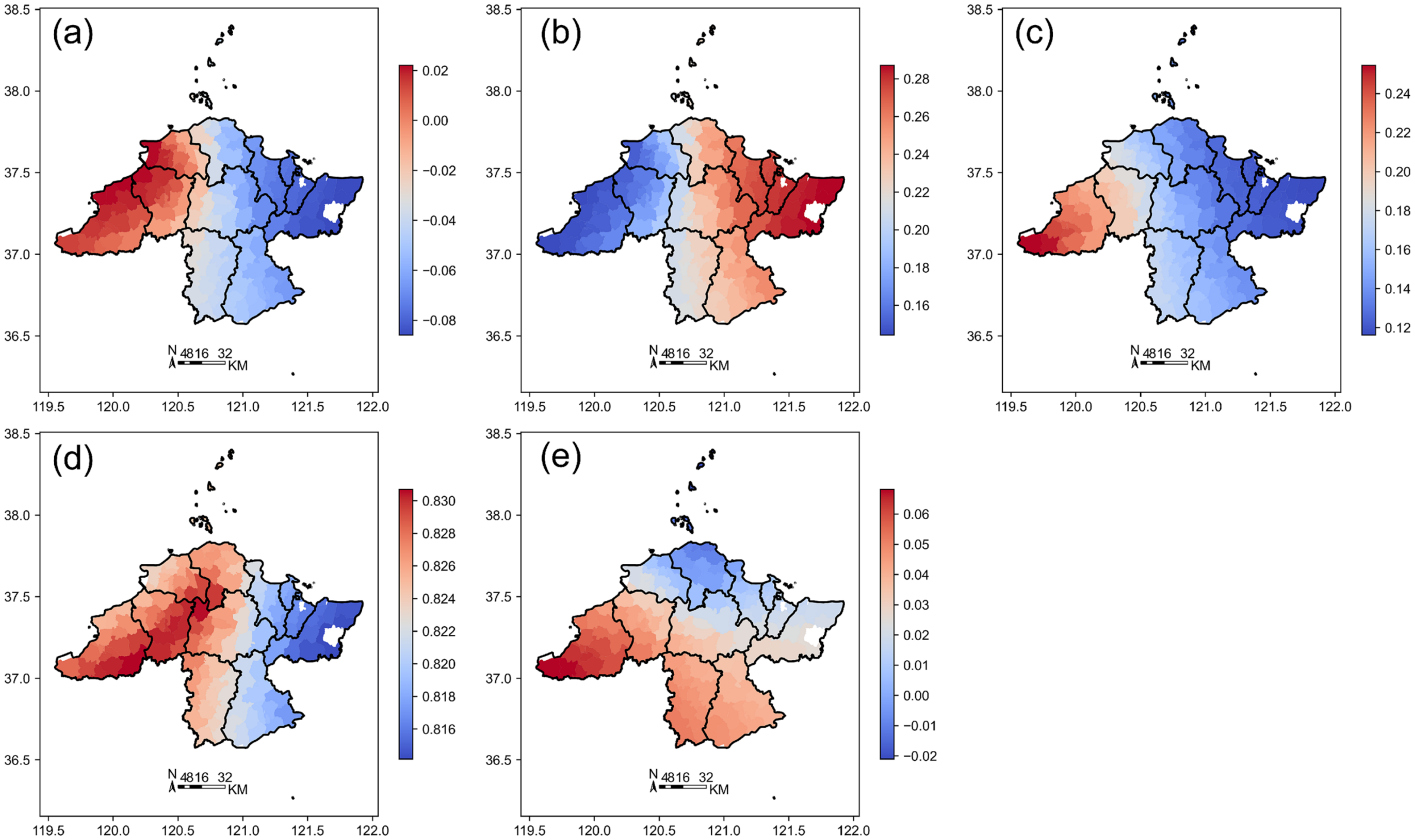
Spatial distribution of GWR regression coefficients in Yantai. **(a)** BCR; **(b)** RD; **(c)** VNL; **(d)** Pop; **(e)** Area. The color gradients indicate the relative magnitude of GWR coefficients, with red colors representing higher coefficient values and blue colors representing lower values. Positive coefficients indicate that the corresponding variable is positively associated with medical facility density at that location, while negative coefficients indicate an inverse association. Larger absolute values denote stronger local effects.

This localized pattern is part of a broader spatially varying relationship between medical facility distribution and multiple driving factors across the city. Figure 9 shows the spatial distribution of GWR regression coefficients in Yantai, displaying local coefficients for (a) Building Coverage Ratio (BCR), (b) Road Density (RD), (c) VNL, (d) Population (Pop), and (e) Area.

Indeed, the effects of independent variables in Yantai display notable spatial divergence, particularly between the western and eastern regions. In the west, higher building coverage, population density, and larger regional area are generally associated with increased medical facility provision. Conversely, in the east, road network density emerges as a more influential factor, positively correlated with medical resource distribution. This east–west contrast underscores the complex interplay of urban form, infrastructure, and socio-economic conditions in shaping healthcare accessibility. By mapping and interpreting this spatial heterogeneity, our analysis provides a nuanced understanding of Yantai’s medical layout dynamics, offering a more precise, regionally tailored basis for future health planning and equitable resource allocation.

#### 4.3.5 Analysis of Weihai results

In Weihai, the impact of variables on medical facilities presents a relatively complex spatial distribution, lacking obvious regularity. The specific analysis is as follows:

(1) BCR (Building Coverage Rate)

In Weihai, BCR has the greatest positive impact in the western part of Rushan City, indicating that areas with higher building coverage are usually accompanied by more construction of medical facilities. This might be related to the increased building density during the urbanization process in Rushan City, driving the demand for medical facilities. However, in the central part of Wendeng District, BCR shows a negative impact, meaning that areas with higher building coverage have relatively fewer medical facilities. This might be due to insufficient allocation or over-concentration of medical resources in some high-density areas.

(2) RD (Road Network Density)

In Weihai, RD shows a strong positive impact in Wendeng District, indicating that increased road network density promotes the distribution of medical facilities. In the eastern part of Rongcheng City, the impact of RD is relatively weak but positive, suggesting that increased road network density in the eastern area has a limited effect on enhancing medical facilities, possibly because the transportation network in this region is not yet fully developed.

(3) VNL (Nighttime Light Value)

The impact of VNL on medical facilities shows relatively extreme spatial heterogeneity, with coefficients fluctuating between −1.0 and 1.0. In a few areas in the southern part of Rongcheng City, VNL shows a strong positive promotion, indicating that areas with stronger nighttime lights are usually accompanied by more medical facilities. This might be related to the high level of economic activity and nighttime social activity in this area. However, on the northwestern fringe of the city, VNL shows a negative impact, indicating that in areas with weaker nighttime lights, the construction of medical facilities is relatively lagging, possibly related to the lower level of urbanization and social activity in this region.

(3) Population (Pop)

Population density has the most significant impact in the eastern part of Rongcheng City, showing a strong positive relationship, indicating that areas with larger populations usually have more medical facilities. This shows that areas with higher population density have greater demand for medical facilities, driving the corresponding resource allocation. In other areas of Weihai, the relationship between population density and medical facilities is relatively weak, showing a slight positive influence.

(4) Regional Area (Area)

The impact of regional area shows a trend opposite to that of population density. In the eastern part of Rongcheng City, the impact of regional area on medical facilities is relatively weak, showing a slight positive relationship, while in the western region, larger areas have a stronger impact on medical facilities, showing a positive effect. However, overall, the impact of regional area is relatively limited and does not significantly drive the distribution of medical facilities.

**Figure 10:**
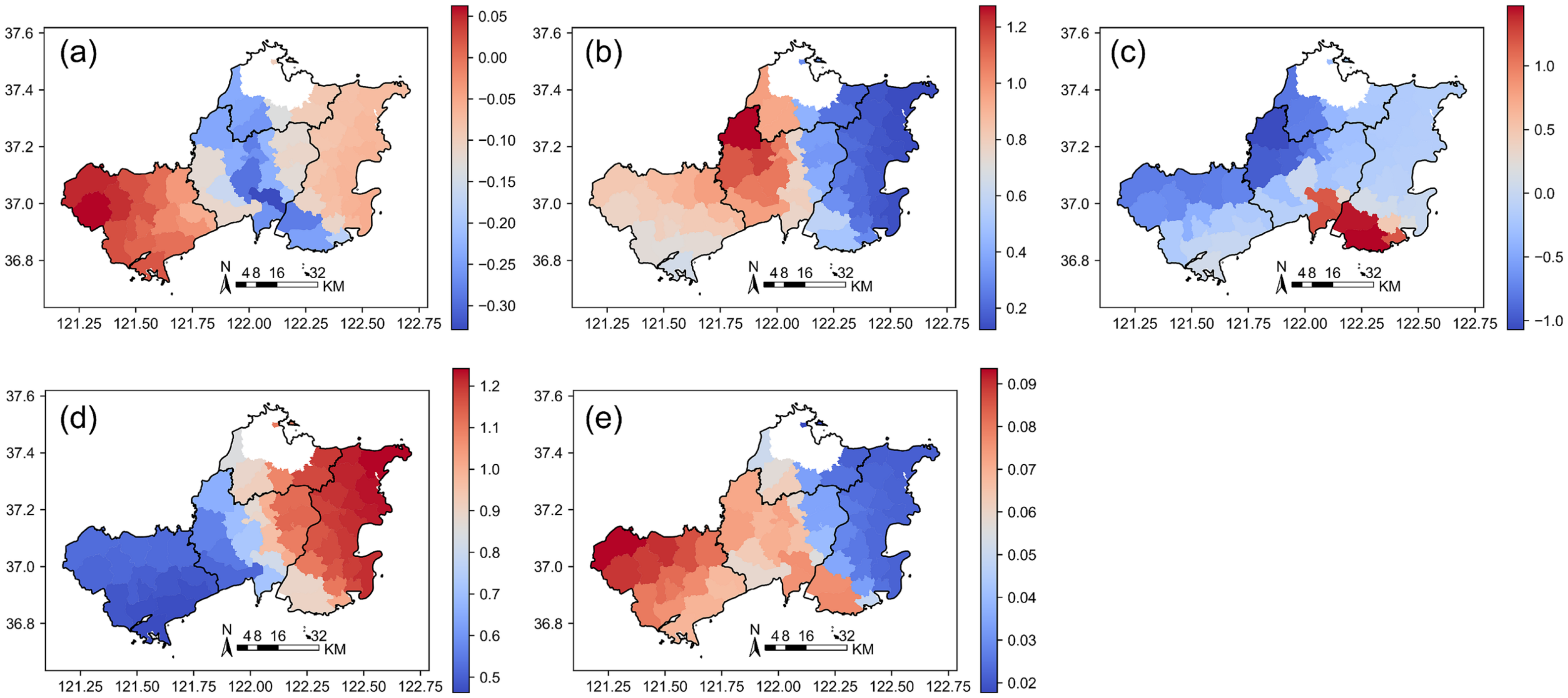
Spatial distribution of GWR regression coefficients in Weihai. **(a)** BCR; **(b)** RD; **(c)** VNL; **(d)** Pop; **(e)** Area. The color gradients indicate the relative magnitude of GWR coefficients, with red colors representing higher coefficient values and blue colors representing lower values. Positive coefficients indicate that the corresponding variable is positively associated with medical facility density at that location, while negative coefficients indicate an inverse association. Larger absolute values denote stronger local effects.

The influence of various independent variables on medical facilities in Weihai exhibits pronounced spatial heterogeneity, with no consistent city-wide pattern. Figure 10 displays the spatial distribution of GWR regression coefficients in Weihai, showing local coefficients for (a) Building Coverage Ratio (BCR), (b) Road Density (RD), (c) Nighttime Light Intensity (VNL), (d) Population (Pop), and (e) Area.

In western Rushan City, higher building coverage and population density are generally associated with greater provision of medical facilities, reflecting typical urban-driven healthcare demand. In contrast, in eastern Rongcheng City, road network density and population density emerge as the dominant predictors, while the effects of nighttime lights and regional area are comparatively weak. These spatial differences highlight the uneven trajectories of urbanization, infrastructure development, and demographic distribution across Weihai’s subregions. Understanding such localized dynamics provides valuable, context-sensitive insights for optimizing the future layout of medical facilities and advancing equitable health service access throughout the city.

## 5 Discussion

### 5.1 Analysis of the causes of medical resource imbalance from the perspective of spatial justice

From the perspective of spatial justice, the allocation of medical resources should be understood not only as a question of numerical distribution, but also as an outcome of how urban space structures access opportunities under specific development trajectories. In this study, spatial justice is employed as an interpretive framework to explain observed spatial disparities, rather than as a directly measured normative outcome.

The empirical results reveal a clear pattern of medical resource concentration in provincial capitals and core urban areas such as Jinan and Qingdao, where Global Moran’s I and LISA analyses identify pronounced “high-high” clusters. In contrast, urban fringes and peripheral zones are characterized by “low-low” clusters, indicating persistent under-provision. These spatial patterns are not solely determined by population size, but are closely associated with urban development intensity, infrastructure conditions, and accessibility constraints.

The GWR results further suggest that population density and road network density exert strong but spatially heterogeneous influences on medical facility distribution. In central urban areas, high population density and economic activity are positively associated with facility concentration, reflecting efficiency-oriented location logic. However, in peripheral and urban–rural transition areas, limited transportation infrastructure and dispersed settlement patterns weaken this coupling, resulting in service blind spots even where population demand exists.

Importantly, this study finds that apparent balance in facility counts does not necessarily translate into equitable access. In cities such as Yantai and Weihai, some high-density communities exhibit nominal medical coverage, yet their effective accessibility remains constrained by spatial dispersion and transportation limitations. This mismatch between service provision and actual access conditions constitutes a form of implicit spatial inequity, consistent with spatial justice theory’s emphasis on substantive rather than formal equality.

Overall, the observed imbalance in medical resource distribution reflects a structural misalignment between urban spatial organization and healthcare service provision. Rather than attributing these patterns to a single causal factor, this study highlights how interactions among population distribution, infrastructure networks, and urban form jointly shape spatial equity outcomes. Spatial justice thus provides a useful lens for interpreting these mechanisms and for guiding future policy efforts toward spatially sensitive and context-aware healthcare planning.

### 5.2 Theoretical implications: how spatial elements reshape the design logic of traditional social policies

For a long time, the formulation of social policies has mostly used administrative divisions, statistical indicators, and population size as the basis for allocation, implying a governance assumption of a “homogeneous space,” i.e., residents in different regions have similar capacity to receive and demand structure for the same policy content. However, this study finds through spatial analysis that spatial elements such as population density, road network structure, and building coverage rate exhibit high heterogeneity within different cities. These differences not only affect the physical distribution of resources but also profoundly affect the possibility of service access and the effectiveness of institutional responses.

From the perspective of spatial justice, social policies that ignore spatial differences are prone to institutional biases that appear equal but are actually unfair. For example, the same number of medical facilities have vastly different service capacities in central urban areas versus urban–rural junctions; in areas with low road network density, even if facilities exist, their actual accessibility is greatly reduced. Traditional policy design often follows the logic of “equal distribution - universal applicability,” while the introduction of spatial factors prompts us to: truly effective social policies should be spatially sensitive strategies of “adapting to local conditions” and “matching to the area.”

Furthermore, the incorporation of spatial elements also suggests that we should shift from a mindset of “static total allocation” to “dynamic spatial adjustment.” For example, the spatial agglomeration or dispersion characteristics revealed by the Gini coefficient and Moran’s Index can serve as a priority basis for resource reallocation; the spatial differences in influence mechanisms identified by the Geographically Weighted Regression model also provide parameter support for refined policies. Spatial data is not only a tool for diagnosing inequality but also “new public knowledge” for reconstructing policy logic and promoting zoned implementation and differentiated governance.

Therefore, future social policies should break through the traditional thinking of “administrative boundary governance” and transition toward “spatial unit responsive governance,” truly achieving geographical justice and institutional fairness in public services. This is not only an application extension of spatial analysis methods but also a redefinition of the value foundation of social policies.

### 5.3 Critical reflection: correcting technocracy

In recent years, research on the distribution of medical resources has increasingly relied on high-resolution data, algorithmic models, and spatial simulations, yielding many “quantifiable and visual” results. However, as criticized by (Rosenberg (44)), over-reliance on technical rationality tends to simplify equity issues into spatial operations such as “location optimization” or “accessibility improvement,” ignoring the underlying institutional arrangements and value judgments.

By introducing spatial justice theory and the Geographically Weighted Regression model, this study attempts to break the “egalitarian trap”—using a single global indicator to represent overall equity, ignoring differences and demands in local areas. The empirical results show that although medical facilities and population show significant coupling in densely populated areas like Jinan and Qingdao, in many areas of Weihai, even with a certain population size or development level, the allocation of medical resources still shows slow response or spatial mismatch. This indicates that resource allocation is not entirely spontaneously adjusted by “demand drive” but may be deeply influenced by institutional factors such as spatial governance logic, policy orientation, and land use competition.

Although this study still primarily uses empirical analysis as its main approach, it incorporates spatial factors that are more consistent with real-world logic in its method design and uses models like GWR to depict the spatial heterogeneity of variables within cities. Compared to traditional global regression methods that treat the entire city as a “homogeneous space,” ignoring the huge local differences in population density and transportation accessibility, our analysis enhances spatial understanding and explanatory power on an empirical basis. Ignoring these spatial characteristics can easily lead to biased conclusions, not only failing to accurately reveal the mechanisms of resource allocation imbalance but also weakening sensitivity and responsiveness to equity issues. However, achieving true medical equity should not rely solely on the “optimal solution” provided by spatial algorithms but must return to the value logic of equity itself, re-examining whether resource allocation responds to the service needs of diverse groups and reflects institutional inclusivity and planning ethics.

### 5.4 Humanistic care dimension: from technical governance to equitable well-being

The ultimate goal of medical resource allocation is to respond to human health needs and the right to survival, not merely to optimize spatial indicators. Therefore, the spatial justice framework should be combined with a “humanistic care” perspective to promote a transition from “quantitative allocation” to “qualitative response.” This study finds that even though Jinan and Qingdao are relatively balanced in terms of facility quantity and layout structure, urban fringe areas and some towns still face practical difficulties in accessing services (45). This is not only a problem of facility density but also a disconnect between the “lifeworld” and “technical governance.”

In this sense, humanistic care requires us to re-examine the ethical boundaries of resource allocation from the “human scale”: Do medical facilities truly respond to community needs? Have marginal areas been neglected for a long time? Has the speed of urban development compressed the space for medical equity? Raising these questions makes the research not only a spatial analysis at the academic level but also a normative diagnosis and ethical intervention serving society.

Meanwhile, the spatial equity evaluation and influencing factor analysis framework constructed in this paper aims to provide local governments and urban planners with actionable assessment tools and intervention basis. We are concerned not only with the current state of resource distribution but also hope to point out future improvement paths for urban decision-makers by revealing the spatial relationships between variables such as population density, road network structure, nighttime lights, and medical resources. For example, road network density not only affects accessibility but also reflects important dimensions of human well-being such as urban spatial organization, convenience of life, and opportunities for social participation. The equity of medical resource allocation is, in the final analysis, the spatial guarantee of human dignity and quality of life.

Based on this, future research should further deepen the theory of medical planning from the perspective of spatial justice, promoting the evolution of urban public service systems toward a more inclusive, sensitive, and caring direction.

## 6 Conclusion

The research approach of this paper also echoes three important issues in health geography (46): first, the spatial accessibility of medical services; second, the impact of urban neighborhood characteristics on access to health services; third, environmental and social justice issues in the process of resource allocation (47). Through spatial autocorrelation analysis, Gini coefficient measurement, and the Geographically Weighted Regression model, this study aims to explore the equity and driving mechanisms of the spatial distribution of medical resources under real-world constraints. The research results show significant agglomeration of medical facilities in core urban areas, service blind spots in fringe areas and urban–rural junctions, and the coupling relationship between inter-regional resource allocation and factors such as population, road networks, and urban structure exhibits significant spatial variability. Specifically, through global and local Moran’s I analysis, we found that medical resources in Jinan and Qingdao are more concentrated and show obvious spatial clustering patterns, while the distribution of medical resources in Yantai and Weihai is more even, but resource shortages still exist in some local areas. Analysis of the Gini coefficient and local Gini coefficient indicates that Qingdao and Jinan have relatively high medical resource equity, while Yantai and Weihai face more serious regional imbalance issues.

At the theoretical level, this study introduces the perspective of spatial justice, pointing out that the equity of medical resource allocation should not stop at the number of facilities or average coverage rate, but should focus on the actual ability and opportunity for residents in different regions to access services. Traditional methods often assume spatial homogeneity within cities, ignoring differences in basic conditions between regions. Through the Geographically Weighted Regression method used in this study, it was found that the influence of various factors varies significantly within cities, indicating that equity evaluation must consider spatial non-stationarity and local differences to more accurately reflect the real pattern of inequality.

Despite its contributions, this study has several limitations that should be acknowledged. First, although the analysis focuses on four representative cities in Shandong Province (Jinan, Qingdao, Yantai, and Weihai), the study does not aim for nationwide generalization. Instead, it emphasizes methodological applicability and context-sensitive insights that may inform similar analyses in other urban regions. In addition, the study relies on open geospatial data sources, such as Amap POI and OpenStreetMap. While these datasets are widely used and relatively stable for formal medical facilities, potential uncertainties related to data timeliness and positional accuracy cannot be entirely eliminated. Second, the explanatory power of the GWR model varies across cities, and relatively lower goodness-of-fit in some cases suggests that additional factors—such as policy interventions, institutional arrangements, and finer-scale socioeconomic characteristics—may further enhance model performance if suitable data become available. Third, this study adopts an explanatory and interpretive approach rather than a causal inference framework. Although spatial disparities in medical resource distribution are discussed in relation to spatial justice theory, specific causal mechanisms (e.g., policy history or land-use regulations) are not empirically identified. Future research could integrate longitudinal data, policy variables, and causal modeling strategies to more rigorously examine these mechanisms.

## Data Availability

The datasets presented in this study can be found in online repositories. The names of the repository/repositories and accession number(s) can be found in the article/supplementary material.
